# Urological research in sub-Saharan Africa: a retrospective cohort study of abstracts presented at the Nigerian association of urological surgeons conferences

**DOI:** 10.1186/1471-2490-13-59

**Published:** 2013-11-14

**Authors:** Jibril Oyekunle Bello

**Affiliations:** 1 Department of Surgery, Urology Unit, University of Ilorin Teaching Hospital, PMB 1459 Ilorin, Nigeria

**Keywords:** Urology, Abstracts, Publication, Peer review, Statistics, NAUS

## Abstract

**Background:**

Nigeria is one of the top three countries in Africa in terms of science research output and Nigerian urologists’ biomedical research output contributes to this. Each year, urologists in Nigeria gather to present their recent research at the conference of the Nigerian Association of Urological Surgeons (NAUS). These abstracts are not thoroughly vetted as are full length manuscripts published in peer reviewed journals but the information they disseminate may affect clinical practice of attendees. This study aims to describe the characteristics of abstracts presented at the annual conferences of NAUS, the quality of the abstracts as determined by the subsequent publication of full length manuscripts in peer-review indexed journals and the factors that influence such successful publication.

**Methods:**

Abstracts presented at the 2007 to 2010 NAUS conferences were identified through conference abstracts books. Using a strict search protocol, publication in peer-reviewed journals was determined. The abstracts characteristics were analyzed and their quality judged by subsequent successful publishing of full length manuscripts. Statistical analysis was performed using SPSS 16.0 software to determine factors predictive of successful publication.

**Results:**

Only 75 abstracts were presented at the NAUS 2007 to 2010 conferences; a quarter (24%) of the presented abstracts was subsequently published as full length manuscripts. Median time to publication was 15 months (range 2–40 months). Manuscripts whose result data were analyzed with ‘beyond basic’ statistics of frequencies and averages were more likely to be published than those with basic or no statistics.

**Conclusions:**

Quality of the abstracts and thus subsequent publication success is influenced by the use of ‘beyond basic’ statistics in analysis of the result data presented. There is a need for improvement in the quality of urological research from Nigeria.

## Background

A major goal of biomedical research is the translation of the discoveries made in the clinic or laboratory into useful measures that decrease disease burden and improves quality of life of target populations. These goals are often achieved by wide dissemination of knowledge obtained from quality research published in peer-reviewed journals with consequent impact on clinical practice [1]. The journals serve, by publishing thoroughly vetted research findings, as both gatekeepers and gateways for the transfer of knowledge amongst health practitioners [2]. Nigeria is one of the top three countries in Africa in research publishing output, though the visibility of Africa’s science is low both on the continent and internationally [2]. Apart from the dissemination of knowledge in the form of published research articles, conferences and meetings are additional avenues for sharing of scientific information usually in the form of abstracts presented at such gatherings. These abstracts do not usually undergo the vigorous peer-review process used by most traditional journals in selecting manuscripts to be published but their unvetted results are communicated to attendees and may influence negatively their clinical practice. An assessment of the content and quality of these abstracts is thus important and gives insights to the validity of contributions made to research at such meetings. The quality of an abstract presented may be judged by its successful (or otherwise) conversion into publication of the full length manuscript in a peer-reviewed journal [3]. The purpose of this study is to determine the characteristics of abstracts presented at the annual conferences of the Nigerian Association of Urological Surgeons (NAUS), the quality of the research as determined by their subsequent publication in peer-review indexed journals and the factors that influence successful publication.

## Methods

### Data collection

All abstracts accepted for presentation at the NAUS conferences from 2007 to 2010 were identified from the conference’s book of abstracts. The characteristics of such abstracts such as year of presentation, type of manuscript, number of authors, area of study, study design/methodology and use of statistics were analyzed. The statistics used in analysis of result data was grouped into ‘basic’ if only frequencies and averages were used; ‘beyond basic’ if statistical tests of significance like Chi-square, Fisher’s exact test, Student t tests, odds ratios, regression analysis etc. were used and ‘none’ if statistics was not used or was not applicable. The use of statistics was described as not applicable for case reports or series and point of techniques. Ethics approval was not required.

**Figure 1:**
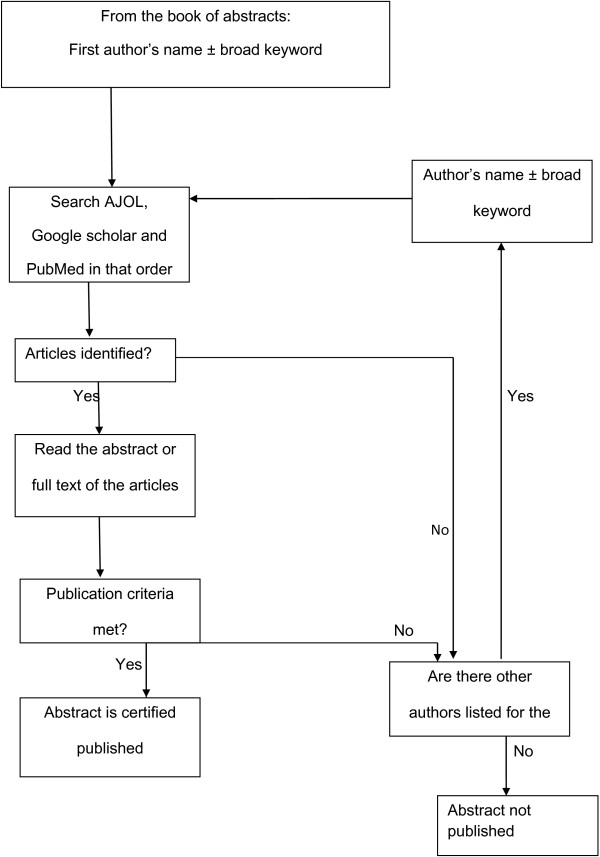
**Search Algorithm.** Schematic diagram depicting the strict protocol used to search for successful publication of full length manuscripts of presented abstracts; AJOL, Google scholar and PubMed data bases were searched until 30th September 2012.

Assessment of successful publication: Subsequent publication of full length manuscripts in indexed journals was assessed by searching through PubMed, Google Scholar and African Journal Online (AJOL) databases using a strict search algorithm adapted from a similar study (Figure 1) [3]. Abstracts were certified as published if 1) at least one author of the presented abstract was included in the final published manuscript and 2) the methodology and result data in the presented abstract is similar to that of the final published manuscript. The databases were searched till 30th of September 2012.

### Statistical analysis

The data was analyzed using SPSS 16.0 statistical software. Chi-square or Fisher’s exact test was used to determine level of statistical significance as appropriate; binary logistic regression was used further to determine the strength of association by analyzing the dichotomous variables of publication and non-publication versus the possible predictive factors. The level of significance was set at p < 0.05.

## Results

Overall, 75 abstracts were presented at the NAUS 2007 to 2010 conferences and all were podium presentations. The median number of abstracts presented yearly was 18 (range; 13–27). Eighteen (24%) of the presented abstracts were subsequently published as full length manuscripts with 2 published prior to presentation at the conferences. The median time to publication for the 16 manuscripts published after presentation at conferences was 15 months (range 2–40 months).

Table 1 lists the absolute number of abstracts presented and the publication rates by conference year. There was a statistical significant difference in publication rate by conference year with the highest publication rate of 50% at the 2008 conference (p = 0.036).

**Table 1:** Number of abstracts presented and their publication rates by conference year

**Conference year**	**Presented abstracts**	**Published manuscripts (% of presented abstracts)**
2007	19	3 (16%)
2008	16	8 (50%)
2009	27	6 (22%)
2010	13	1 (8%)
Total	75	18 (24%)

Table 2 lists the number of presented abstracts by abstract characteristics (type of manuscript, study design/methodology, area of study and use of statistics) and provides a comparison of the proportions of the abstracts which were subsequently published as full length manuscripts. When evaluated with respect to the type of manuscript, 33% of all presented abstracts were case reports or case series while the remainders were original articles; 20% of the case reports and case series were published while 26% of the original articles got published. There was no statistical significant difference in publication rates by type of manuscript (p = 0.778). An analysis of the study design/methodology of study used in the presented abstracts revealed retrospective studies were commonest. There was no statistical significant difference in publication rates by the type of methodology used (p = 0.204). The area of study of the presented abstracts were also analyzed; most of the abstracts were on adult urology (66, 88%), only a few (6, 8%) were on paediatric urology and none were investigative urology (Basic science) research.

**Table 2:** Presented abstracts (N = 75) and published manuscripts (N = 18) by abstract characteristics

	**Presented abstracts**	**Published manuscripts**	
**Abstract characteristics**	**n,% presented abstracts**	**n,% characteristic category**	**p-value**
Type of manuscript			0.778^1^
Case report	18(24%)	4(22%)	
Case series	7(9%)	1(14%)	
Original articles	50(67%)	13(26%)	
Study design/Methodology			0.204^1^
None^3^	27(36%)	5(19%)	
Retrospective	31(41%)	9(29%)	
Prospective	14(19%)	2(14%)	
Cross sectional	3(4%)	2(67%)	
Area of study			0.839^2^
Adult urology	66(88%)	15(23%)	
Paediatric urology	6(8%)	2(33%)	
Others	3(4%)	1(33%)	
Use of statistics			0.009^1^
Basic	41(55%)	8(20%)	
‘Beyond basic’^4^	7(9%)	5(71%)	
None	27(36%)	5(19%)	

^1^Chi square test.

^2^Fisher’s exact test.

^3^Case reports, case series, point of technique.

^4^Statistical measures beyond the basic measures of frequencies and averages i.e. tests of significance, logistic regressions.

The use of statistics in analysis of result data presented in the abstracts was analyzed. Table 2 also shows the proportions of presented abstracts and publication rates by use of statistics. There is a statistical significant difference in publication rate by use of statistics in result data analysis; over 70% of abstracts using ‘beyond basic’ statistics are published as full manuscripts while less than 20% of abstracts with only basic statistics or none at all are similarly published (p = 0.009). Binary logistic regression analysis was conducted to predict subsequent publication of presented abstracts as full manuscripts using ‘use of statistics’ as predictors; a test of the full model against a constant only model was statistically significant, indicating the use of statistics reliably predicts abstracts which will be subsequent published as full manuscripts (Chi-square = 7.939, p = 0.019, df = 2). The Wald criterion and Exp (B) demonstrates that only use of ‘beyond basic’ statistics significantly predicts subsequent successful publication and are 11 times more likely to be published (Wald = 6.082, Exp (B) = 11.000, p = 0.014).

Multiple authored manuscripts formed the larger proportion of the presented abstracts (47, 82.5%). There was no statistical significant difference in publication rate between single authored and multi-authored abstracts (p = 0.056).

Majority of the published abstracts (14, 78%) were in local or national journals with the remainder in international journals.

## Discussion

The annual NAUS conference is hosted usually in November of every year by a different Nigerian city and it is a gathering of a few scores of urologists and allied health care practitioners from all around the country and sometimes abroad. The conferences are significantly smaller than the large urological mega-conferences in Europe and North America in both number of attendees and the absolute numbers of abstracts presented; the median number of 18 abstracts presented yearly at NAUS is far less than the several hundred often presented yearly at the mega international urological conferences [3,4]. Due to these differences, the international impact of the abstracts presented at NAUS conferences would be comparatively smaller than those of the international conferences; however for the local attendees the information disseminated may influence their subsequent clinical decision making and thus, assessing the quality of these abstracts is of utmost importance.

**Figure 2:**
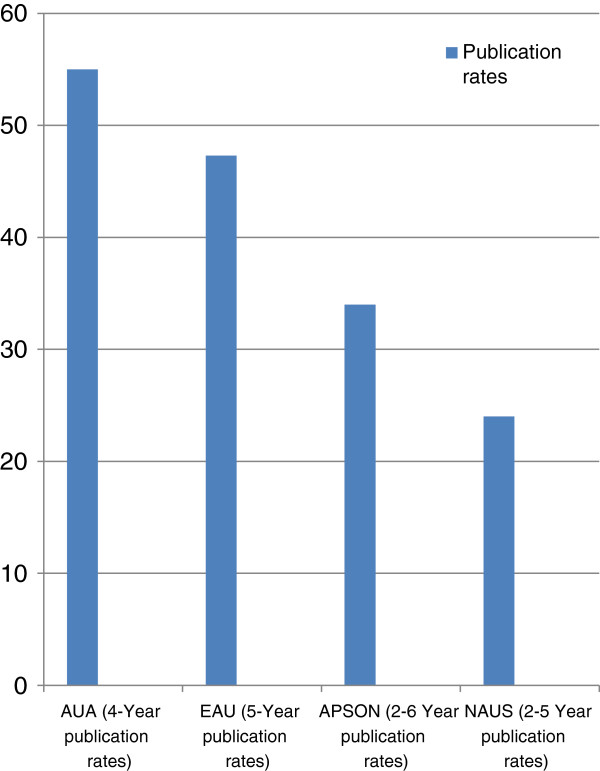
**Publication rates.** Bar chart comparing the publication rates of full length manuscripts for abstracts presented at biomedical conferences. AUA, American Urological Association; EAU, European Association of Urology; APSON, Association of Paediatric Surgeons of Nigeria; NAUS, Nigerian Association of Urological Surgeons.

The subsequent publication rate of abstracts presented at biomedical meetings has been used to assess the quality of abstracts presented at such meetings [3,4]. The finding in this study of 24% publication rate for presented abstracts in peer reviewed journals is lower than figures reported for two major international urological conferences but similar to the 34% reported for the conference of the Association of Paediatric Surgeons of Nigeria [3-5]. This may be attributable to the fact that these international meetings are bigger, more prestigious and thus attracts better quality abstracts with subsequent higher publication rates. There may be other factors at play, as reports on other large, international and prestigious urological conferences have shown publication rates similar to that found in this study [6,7]. However, it may be inappropriate to make these comparisons of publication rates as the figures reported are not standardized and range widely representing two to six-year publication rates (Figure 2). The period of observation for publications was only 2 years for the 2010 conference which is shorter than the periods observed for publications for the 2007 to 2009 conferences; this may account for the low publication rate (8%) observed for the year 2010 in this study. Several studies have looked at the reasons for subsequent non-publication full length manuscripts of abstracts presented at meetings; the causes are multiple and include anticipated rejection, low prioritization and ‘lack of time’ by authors to prepare manuscripts [1]. There may be additional reasons in Sub-Saharan setting which may include poor healthcare infrastructure and hostile research environment with very little funds made available. Nigeria also has very few and over-stretched urology specialist man-power with the ratio of urologists to the Nigerian population estimated to be 1: 3.8 million [8]. This is unhelpful as the over-burdened urologist may find it difficult to devote adequate time to research. However, despite these impediments, Nigeria remains one of the top three countries in Africa in science research publication [2].

Result sections of manuscripts often uses various forms of statistical analysis to present data obtained from studies conducted. This may be in form of basic statistical measures of frequencies and averages or use of a wide range of ‘beyond basic’ statistical analysis of data. The ‘beyond basic’ statistical measures includes tests of statistical significance like Chi-Square, Student t tests, regression analysis and a host of other tests; its use improves the level of evidence in manuscripts [9]. The use of statistical measures is usually not applicable for case reports, case series and point of technique (a description of surgical technique). Analysis of abstracts presented at NAUS revealed that the majority of abstracts used only basic statistics (55%) and for another large group (36%) no statistics were used or its use was not applicable. This poor use of statistics may have effectively lowered the quality of information presented in such manuscripts; this study’s finding that use of ‘beyond basic’ statistics significantly improves the odds of publication of a full length manuscript lends some support to this. It is not surprising that medical practitioners and researchers find learning statistics difficult and there are evidences of widespread misuse of statistics in published manuscripts [9]. Training of medical personnel at both undergraduate and postgraduate levels in statistical concepts and methods has been suggested to improve manuscript writing and the comprehension of statistical contents of published articles [10,11]. None of the abstracts studied were on basic science or investigative urology and most were also retrospective studies and case reports. Though these factors do not significantly affect the odds of subsequent publication of full length manuscript in this study, others have reported that abstracts on basic science (as opposed to clinical research) are more likely to be published in peer-reviewed journals [1].

The median time to publication of full length manuscripts of abstracts presented at NAUS conference is 15 months and this is similar to those reported for the mega conferences [3,4,7]. This suggests that quality manuscripts undergoing traditional peer-review process will take averagely similar times to get published. Time interval between the conference presentation and submission of the complete work to a journal would however have an effect on this. This study also found that most of the published manuscripts were in local or national journals; this may probably be the result of the manuscript contents being mostly relevant locally and would therefore be more readily accepted by the journals. A report mapping biomedical research publishing characteristics in Sub-Saharan Africa concluded that most authors in the region publish in international journals [12]; their finding may be skewed by the methodology used as only MEDLINE indexed journals were searched, ignoring African Journal Online (AJOL) which has a large online collection of African journals. It is difficult to ascertain the differential impacts on Nigerian urological practice of publishing in local/national rather than international journals as local/national journals are not necessarily easier to access than international journals by local urologists in this age of improved internet availability; the scientific content in local/national journals may however be of poorer quality [13]. A study on the level of evidence in manuscripts published in five major Nigerian journals from 2005–2006 revealed that approximately 90% either had no evidence at all or only level 4 evidence and none had level 1 evidence [13]. This suggests a majority of the published manuscripts in the National journals are perhaps of doubtful value to medical practitioners and researchers.

Three of the presented abstracts in this study were published as full length manuscripts before the meetings. This has been observed in similar studies of urology meetings and can be regarded as unhelpful to scientific discourse at such gatherings as it goes against the principle that presented abstracts at meetings should be recent unpublished works [3].

Limitation of this study includes that only PubMed, AJOL and Google Scholar were searched. Manuscripts published but not indexed in these will be missed and this is not unlikely in Nigeria with many un-indexed journals [14]. A survey of 158 medical journals published in 33 African countries showed that most had circulations < 1000, published 4 or fewer issues a year and are not included in major bibliographic indexes [14].

## Conclusions

The NAUS annual conferences are comparatively smaller than similar International urology meetings in number of abstracts presented and only a quarter of the 75 abstracts presented at the 2007 to 2010 conferences were subsequently published as full length manuscripts in indexed journals. The use of ‘beyond basic’ statistics in analysis of results data is low in the presented abstracts and its use improved the odds of subsequent successful publishing of full length manuscripts. There is need for improvement in the quality of research work done in this part of the world and scientific committees may need to be more rigorous in evaluating submitted manuscripts.

## Abbreviations

NAUS: Nigerian association of urological surgeons; AJOL: African journal online.

## Competing interests

The author declares that there are no competing interests.

## Pre-publication history

The pre-publication history for this paper can be accessed here:

http://www.biomedcentral.com/1471-2490/13/59/prepub
